# “A Ronin Without a Master”: Exploring Police Perspectives on Digital Evidence in England and Wales

**DOI:** 10.3390/bs15101416

**Published:** 2025-10-17

**Authors:** Magdalene Ng, Rachael Medhurst, Coral J. Dando, Ray Bull

**Affiliations:** 1 Psychology, School of Social Sciences, University of Westminster, London W1W 6UW, UK; 2 Faculty of Computing, Engineering and Science, University of South Wales, Newport NP20 2BP, UK; 3 Psychology, School of Science, University of Derby, Derby DE22 1GB, UK

**Keywords:** police officers, digital evidence, decision-making, investigation, investigative interviewing, court

## Abstract

Despite digital evidence (DE) now being a major component of most criminal investigations, very few studies have examined how police officers themselves evaluate and use DE over the course of an investigation. Drawing on in-depth interviews with *N* = 13 police officers from England and Wales, four themes are presented: (i) Sense-making and handling of digital devices and DE in investigations; (ii) The interpretation and reliability of DE; (iii) Strategic use of DE in investigative interviews with suspects, with a subtheme of Digital devices and DE in victim-centered interviews; and (iv) DE in the courtroom. While often seen as objective and infallible, DE is fragile, volatile, and legally complex, highlighting the cognitive and interpretive work that officers must do when dealing with DE. This is important because this work has a direct impact on how investigations proceed, including what is taken from crime scenes and how it is used in investigative interviews. Findings show how DE creates unique challenges and opportunities within investigative interviewing, extending research on strategic disclosure into the digital domain. Future directions include setting up better communication workflows to reduce epistemic drift and offering more DE interpretation training to help officers in an increasingly digital environment.

## 1. Introduction

Very few crimes are investigated in today’s criminal justice system without the involvement of digital devices (NPCC, 2020). Digital devices include mobile phones, smart appliances such as smart refrigerators and smart air fryers (Maxwell, 2023), home assistants such as Alexas (Krueger & McKeown, 2020), fitness trackers (Kang et al., 2020), USBs, CCTV, and point-of-sale (POS) devices (Frisby et al., 2012; Ng et al., 2024). These devices can store or create digital evidence (henceforth referred to as DE). Mobile phone data, call records, geolocation, online activities, vehicle telematics, router and server logs, and cookies or cache data are a few examples of DE (Jones & Brookman, 2024b).

### 1.1. From Crime Scene to Courtroom

The standard digital forensic process across the investigative chain begins with the identification and collection of digital devices, all the way through to the final presentation of analysed DE in court. This process usually involves multiple actors along the investigative cascade, going through several handovers. At the start of this chain, police officers (and sometimes scenes of crime officers, also known as SOCOs) seize digital devices suspected to contain data of evidential value. After seizure, they would be placed into Faraday bags or shields to be preserved (Lennox-Steele & Nisbet, 2016). Larger devices such as vehicles with telematics are usually transferred to a secure facility to be stored and later analysed (College of Policing, 2022).

After collection and preservation, DE is handed over to digital forensic investigators (DFIs) for analysis and extraction, which calls for specialised techniques (Ng et al., 2024). A digital media investigator (DMI) is sometimes involved, serving as an intermediary between police officers and DFIs (Wilson-Kovacs, 2021). Not all data from a device is immediately usable as evidence. Only once data is extracted, preserved, and analysed using validated forensic methods that meet courtroom standards does it become digital forensic evidence. It is then passed back to investigating officers to be used in the investigation (Casey, 2019). Investigative interviewing is a key part of the investigation process, which we will expound on below (Holmes, 2024).

### 1.2. The Unique Characteristics of DE

DE has distinctive characteristics that set it apart from other forms of evidence, such as traditional evidence (see Table 1 for further delineation of these differences).

Volume. To put things in perspective, a single mobile phone can hold up to 33 million paper pages after extraction, substantially surpassing the volume produced by traditional evidence such as fingerprints or DNA (GovNet, 2025). Mobile devices can yield all sorts of data, such as text messages, call records, images, GPS traces, and application activities; all this data provides detailed insights into an individual’s movements, behaviours, and intentions (Jones & Brookman, 2024b), far more than, say, a single strand of hair.

Highly volatile and mobile. Unlike traditional evidence, DE is inherently volatile, where volatility refers to how long the data will remain available. It is also highly mobile, which means it is easily transferred and tampered with (Casey, 2019) such as via remote wiping. This can mean that tracking, authenticating, recovering, and reconstructing its timeline becomes complex and ambiguous. Not all digital data and sources have the same lifespan; some are more likely to be volatile (and therefore more ephemeral) than others and will require prioritisation during seizures and triage (Henry, 2009; United Nations Office on Drugs and Crime, n.d.). For example, remotely logged data, routing table, ARP cache, process table, and kernel statistics are more volatile compared to CPU, cache, and register content which are more stable, and therefore should be prioritised first.

Encryption and obfuscation. A normal inquiry can involve up to five devices, some often being encrypted (GovNet, 2025). As a result, officers often find it difficult to gain access into devices such as mobile phones without the appropriate passwords or decryption keys (Jones & Brookman, 2024b). Obfuscation refers to techniques that make digital data harder to detect by the use of hidden folders, steganography, and vault applications (Zhang et al., 2019). Furthermore, what and how much DFIs can extract depends on certain factors such as phone brand, operating system versions, and device security features. For example, Apple devices are known for their strong encryption and built-in security measures, which can significantly limit what digital forensic specialists can access.

### 1.3. The Present Study

Despite its current central role in almost every criminal investigation and unique attributes, little is known about how officers actively interpret and make decisions with regard to DE across the investigation lifecycle (Brookman et al., 2020). Decisions about what to seize, how to interpret it, and how to disclose it in interviews or present it in court can no doubt influence investigative outcomes. Although investigative interviewing is a well-researched area (Bull & Blandón-Gitlin, 2019), its relation to DE remains under-researched compared to traditional or witness evidence (Clemens et al., 2010; Smith & Bull, 2012; Wells & Loftus, 2003). While some initiatives have acknowledged this (e.g., Bull & Milne, 2020; Dando, 2020; ICO, n.d.; NPCC, 2024; Polman et al., 2024; Tekin et al., 2015), few examine how officers understand and use DE to interview suspects and victims in practice.

Sensemaking is the process whereby individuals make meaning in unexpected or ambiguous situations (Weick, 1995). In policing, sensemaking takes shape where officers interpret new situations, make sense of cues, and act (van Hulst & Tsoukas, 2023). Sensemaking (e.g., Innes et al., 2021; Jones et al., 2021) and epistemological perspectives emphasise that the interpretation of DE must be made sense of, rather than taken as self-explanatory (Buscariolli, 2023). In view of this, our main research questions are as follows:

1. How do police officers define and understand digital devices and DE within investigative practice?
2. What guides their decisions around the relevance of digital devices and seizing practices?
3. How is DE used in suspect and victim interviews?
4. How do officers perceive standards, reliability, and courtroom admissibility regarding DE?

## 2. Materials and Methods

Participants. *N* = 13 police officers from forces across England and Wales were recruited using a combination of snowball and expert sampling. Recruitment involved directly reaching out to professional contacts and via LinkedIn. Participants represented a range of forces, including the Metropolitan Police Service, West Midlands Police, and the British Transport Police. Participants’ ages averaged 39.4 years, ranging from 22 to 53 years of age. The sample included 11 male and two female officers, all with varied levels of experience, among them several DMIs who are also sworn officers. Their perspectives were included to ensure that this study captured the breadth of police practice with DE, consistent with other studies in the field (Brookman et al., 2020; Wilson-Kovacs, 2021).

Materials. Participants engaged in semi-structured interviews developed through collaboration and discussions among all authors (see Supplementary Materials). This interview protocol covered participants’ roles, their understanding of both “digital devices” and “DE”, what guides them in searching and seizing DE in crime scenes, and the use of DE in interviews with suspects and victims. Participants were also asked about their perceptions of the standards, reliability, and admissibility of DE. The semi-structured format allowed officers to share their experiences freely, while also keeping consistency across interviews.

Procedure. All participants received an information sheet in advance, outlining the study’s aims, their right to withdraw, and assurances of confidentiality and anonymity before giving their consent to participate (see Supplementary Materials). Interviews were conducted by the first author and audio-recorded with consent on Microsoft Teams and later transcribed. To ensure anonymity, pseudonyms were used and all identifying information was removed during transcription. The interviews took an average of one hour, eight minutes, and 43 s, ranging from 33 min and 53 s to one hour, 31 min and 42 s. After each interview, participants were provided with a written debrief and the research team’s contact details (see Supplementary Materials). No financial incentives were provided.

Reflexive thematic analysis. Interview data were analysed post-transcription using reflexive thematic analysis, following the stages outlined by Braun et al. (2023). This process was an iterative one which involved many rounds of coding, reviewing, and refining. This leant towards a deeper understanding of the rich dataset. While the interview protocol did not directly ask about courtroom practices, participants often raised issues about how DE is admitted and presented in court. These accounts were coded inductively and developed into a key theme as will be discussed in the next section (Byrne, 2022). NVivo 14 was used to manage coding and organise the dataset, and a reflexive statement is included in Appendix A.

## 3. Results

**Figure 1:**
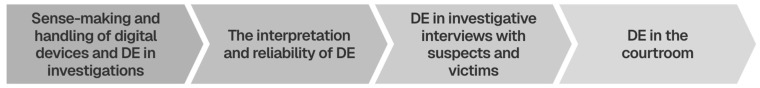
Thematic map of DE across the investigative lifecycle.

The analysis yielded five overarching themes. The fifth theme, which addresses future trajectories of DE and emerging technologies, is beyond the scope of this article and will be reported elsewhere. In this paper, we present four key themes: (i) Sense-making and handling of digital devices and DE in investigations, (ii) The interpretation and reliability of DE, (iii) Strategic use of DE in investigative interviews with suspects, with a subtheme of Digital devices and DE in victim-centred interviews, and (iv) DE in the courtroom. The types of crime reported by participants in our sample range from domestic abuse, homicides, robberies, terrorism, to crypto farms for criminal profit. Figure 1 illustrates the thematic map across the investigative lifecycle.

### 3.1. Theme One: Sense-Making and Handling of Digital Devices and DE in Investigations

Participants vary in their understanding and definition of “digital devices” and “DE”. Participants without specialist digital knowledge show less familiarity with both terms, conflating one with non-digital tools that happen to be electronic (e.g., breathalysers, drug tests, and fingerprint machines):

“Digital… I don’t really know how to explain it. Like phones and stuff, bank cards… things that connect to the Internet. It’s not in front of you… it’s connected to something. And then evidence kind of goes off… like phones, banking, tracking devices.”

(CD)

Some participants may also overlook the fact that not all DE comes from “smart” or connected devices:

“[Digital devices are] electronic devices usually that use communication systems, that might use the Internet, might use radio signals. That’s usually my understanding of digital device and the content held within those devices could be considered digital evidence.”

(BL)

In drug investigations, for example, “burner phones” without internet or location capabilities are routinely seized for call logs or SMS content (Hernandez, 2025). Similarly, USB sticks, SD cards, and external hard drives can also be examined despite their lack of internet connectivity, as they may hold valuable evidence. This highlights a broader issue, that participants without specialist technical knowledge frequently associate “digital” with connected or cloud-based technology, when in fact it includes any device capable of storing, transmitting, or processing electronic data whether online or offline.

This conceptual uncertainty can carry over into practice, in shaping how a device is seen as evidence, how it is handled, prioritised, and if it is seized at all in crime scenes. When officers are unfamiliar with DE or certain types of it, they may choose not to follow digital lines of inquiry at all, putting it in the “too difficult box” (WS). This can imply missed evidence:

“Do I think we are missing evidence? Yes, I think you’d be very naive to… to say that we weren’t.”

(SK)

Participants note that judgments of relevance to seize are often based on device familiarity, as well as availability, and access (e.g., the British Transport Police’s advantage of having station-wide CCTV infrastructure). This points to officers using sense-making under constraint (Simon, 1972; Weick, 1995) to make real-time judgments of what digital devices ‘matters’ in an investigation based on what they can immediately see, access, and readily understand within ambiguous environments.

Most crimes almost always lead to the seizure of mobile phones (TS). TS described how discovering an “ethnic cleansing book” during a search prompted her to seize phones and AirTags, which highlights how certain items and discoveries can shift investigative framing and how digital devices can be reclassified as potential evidence and change in meaning (Elzinga, 1997).

Participants routinely describe only having small windows to seize digital data that is time-sensitive and volatile (e.g., CCTV systems), naming it the “golden hour” period:

“The golden hour… is like a set time period where if it goes to court, you’ve got a higher chance of prosecution if you seize it within that amount of time… Onboard CCTV only lasts for seven days.”

(CD)

Similarly, they also describe challenges in gaining access to cloud data before token expiration. Tokens are access to applications (temporary access keys) to cloud-based data that often expire within a matter of days or weeks (Grassi et al., 2017). This design feature, intended to provide security and privacy for users for devices (e.g., laptops, cloud-connected mobile phones, biometric access controls on devices, encrypted storage media, Internet of Things technology using encrypted data transmission), is especially complex when legal workarounds are difficult to process or unlikely to happen.

“If you get that device within the time that the tokens on the phone are still in date… that’s a big challenge. Mobile phone backlogs are quite significant, nine to twelve months in some places… and even three months nationally can mean token expiry… If you miss that opportunity, then you’re going to go through expensive legal treaties, which most of the time don’t get done.”

(NC)

UK policing guidance, now overseen by the National Police Chiefs’ Council (NPCC), advises that officers should not interact with suspects’ live devices wherever possible, as doing so risks altering data (ACPO, 2012). In practice, however, officers describe situations where they feel pressured to search through a device immediately to retrieve volatile information (e.g., making on-the-spot attempts to access a suspect’s phone using a guessed password before the data disappears) before formal seizure. Participants often describe relying on their “personal judgment” (CL, BL) and many give accounts of justifiable decision-making, sometimes risking not being able to convict a suspect in order to prioritise safeguarding victims. JK describes this deviation as akin to being “a ronin without master”:

“Technically not allowed, but sometimes you have to weigh up the risk. If that phone locks, I’m not going to get the evidence… We get that sometimes with high-risk offences where we’re not as bothered about whether we get a successful criminal conviction as about safeguarding someone.”

(NC)

“You just need to put 5555 [as the phone’s pin] in just to quickly access the message.”

(MS)

Notably, participants mention that law enforcement is increasingly generating their own DE through technologies such as body-worn cameras (BWCs) (“Body worn is an amazing piece of, you know, evidence we have”, TS), live facial recognition (LFRs), and video-recorded interviews (VRIs) (DB and TS). VRIs are interviews conducted with vulnerable witnesses such as children or intimidated victims that are formally recorded under Achieving Best Evidence guidance (Ministry of Justice, 2023). It is particularly valuable in capturing the accounts of vulnerable witnesses, as its format minimises trauma and allows them to share experiences that could be otherwise difficult for them to articulate.

Taken together, this theme helps explain why DE is handled inconsistently: not out of neglect, but often due to cognitive overload, time pressure, digital illiteracy, and safeguarding considerations. These early-stage uncertainties also illustrate practical sense-making under constraint, setting the conditions for interview preparation (Simon, 1972; Weick, 1995) and shaping how, and whether, DE is later deployed in investigative interviews.

### 3.2. Theme Two: The Interpretation and Reliability of DE

DE can be highly reliable, but participants in this study underline that its reliability depends on several key points in the investigative process. After DE is extracted by DFIs, investigating officers are typically handed back its extracted form on a medium such as “a USB stick” (BL), together with a digital forensic report. Officers must then interpret this data. Participants highlight that there is often a struggle at this critical juncture for them to understand, interpret, or act on it meaningfully. Some note that digital extractions often result in a “data dump to people who don’t understand what it means” (NC):

“You can stick a mobile phone or a computer through the best lab in the in the world. If you then give it to someone who doesn’t have a clue what it means, the risk is there because it’s not the extraction report that’s going to court.”

(NC)

This introduces the concept of *epistemic drift* (Elzinga, 1997). Applied to DE, the drift here being how the meaning of DE changes or is lost as it passes through different actors across the investigative chain. CL describes how officers often try to “get to the needle in a haystack”, a metaphor for the challenge of officers needing to identify relevant material from a sea of data. The difficulty in locating this so-called “silver bullet” directly affects their ability to use DE effectively in investigative interviews:

“[We are] handed a USB stick full of information… and then what? The trouble is getting into that needle to be able to ask the right questions. You’ve got the evidence. But how do you make it usable? Understandable? Actionable?”

(CL)

“Somewhere in there, something there might be one single line or single word. That is, that is that golden nugget… that silver bullet for that investigation.”

(WS)

Many officers do not receive training on how to interpret this data, with the quote below highlighting the disconnect between data extracted from a device and what officers can understand or meaningfully use:

“The issue between the extraction and maybe what the investigation officer gets as a product I don’t think is very well explained. That’s one of the biggest threats to digital investigations, the lack of understanding by the individual reviewing the product.”

(SK)

Importantly, participants acknowledge the presence of bias in DE. BL notes that “like any human being, we can suffer from tunnel vision”. NC states that sometimes they “just report on the cell site that covers the location of interest… even if there’s fifty that don’t.” This can lead to misinterpretation (e.g., IP logs, router data, location signals):

“It’s unconscious bias because when we are used to the way that we look at [for example], child sexual offences online, we would do IP address stuff, so we would either go to a piece of software which would tell us who’s been uploaded in DMS to the children via IP address.”

(NC)

Notably, participants reveal how the rise of deepfake technologies, synthetic audio, and other AI-enabled crime is decreasing confidence in the reliability of DE while impacting speech and audio forensics:

“We’ve seen fake CCTV. You might get a call in your son’s voice saying ‘Hi Dad…’ and believe it’s real… I could generate your voice with 30 seconds of audio.”

(WS)

Another participant recounts a case in which a suspect admitted a CCTV image resembled him, but still denied it was him, illustrating a case of the *liar’s dividend* (Chesney & Citron, 2019). This is where even real DE can now be dismissed by suspects as fake evidence, creating new challenges for police and courts particularly when investigators and courtroom actors lack the literacy to counter such claims. The same participant reflected on his own fallibility when he misidentified his own daughter in a school play:

“I had one suspect say to me… “Officer, I agree that CCTV image of me from that CCTV… it really does look like me but… it’s not me. I saw my daughter perform on stage and I said to my wife ‘Look at our daughter. Isn’t she doing well?’. And my wife said to me ‘That’s not your daughter’.”

(GO)

MS points out that not all DE is equal in its complexity. While basic digital data such as text messages or social media posts is easier to understand, there are more complex investigations involving network artefacts (e.g., IP tracing and routers) or cyber-enabled abuse cases (e.g., impersonation and website creation) that require a deeper understanding of how the internet works:

“Somebody creating fake accounts of different platforms, social media platforms and adding a person to monitor and keep tabs and store the evidential threshold for that becomes a lot more complicated because then you’re looking at IP addresses, Internet service provider data logons.”

(GO)

Furthermore, some data types are inherently more accurate than others (e.g., GPS within 10m, whereas IP, cell site, and router logs are more ambiguous):

“Cell site evidence, is a bit different because that’s still a bit grey.”

(NC)

To make matters worse, some participants appear to over-trust the reliability of DE (“It’s probably 100%”, RP), treating it as ‘black and white’ or self-evident. Treating all DE with the same level of confidence can lead to errors and misinterpretation (e.g., IP address confusion, router log misinterpretation). This can lead to missed or unreliable evidence and even wrongful convictions, making it especially dangerous:

“There’s been a general attitude… that you know, computers are infallible and the digital records are perfect.”

(JK)

“All of a sudden people are getting labelled paedophiles and all that kind of stuff when actually they’ve done nothing wrong.”

(NC)

This theme underlines that the reliability of DE is not only technical but sociotechnical. There are challenges to its interpretation and reliability that are magnified once this evidence enters the courtroom, as explored in Theme four.

### 3.3. Theme Three: Strategic Use of DE in Investigative Interviews with Suspects

This theme delves into how participants strategically use DE during suspect interviews, focusing on the disclosure of DE and suspect reactions, especially in sexual offenses. Participants report that the usual (phased) structure of these interviews is to first establish attribution (that is, showing that digital activity comes from the suspect). DB illustrates how this is established across multiple synced devices with the example of an upskirting case:

“All upskirt images I found were found on each device because they’re all communicating with each other, I attributed the digital device to him. So I went and done a request to his network provider who said this phone belongs to himself. I’ve got the longitude and latitude and found the exact locations where it was all happening… these images weren’t downloaded and were taken from the device, which is very key.”

(DB)

These directly shape the types of questions asked during interviews. Importantly, CL points out the danger of officers “not asking the right questions” or not explaining DE well due to a lack of understanding of digital forensic reports. Sometimes, officers may rely too heavily on “smoking gun” signals like location pings or IP logs, and give more weight to traditional evidence over DE because it is seen as too complex or unfamiliar.

Suspects may also struggle to understand technical details. For the benefit of both officers and suspects, DE should be explained in plain, everyday language, and supported with visuals (e.g., annotated screenshots, timelines) where possible, representing an extra challenge compared to traditional evidence such as DNA and fingerprints:

“Really important that you present it to the suspect in a way that they understand it… to be honest with you, I would struggle to explain a lot of it.”

(GO)

Visual formats like PDFs, laptop displays, and printouts are typically used as exhibits, highlighting the importance of presentation formats when it comes to DE.

“With drug dealing, messages are important. We would usually have a printout of a series of messages and say to the suspects, ‘These are the messages that we believe that you have sent’.”

(BL)

As prior research shows, techniques such as unanticipated questions are only effective if interviewers understand the material well enough to use them strategically (Vrij et al., 2011). Cognitive-load approaches to interviewing will be more challenging to apply with DE if officers often struggle to interpret or translate DE into plain language:

Participants vary in when they tend to disclose DE, often tailoring their approach to the type of offence. In indecent image cases, for example, IP explained that they “don’t put much to them at the start” but instead allow an initial account before confronting them later.

“Evidence would be used, more often than not, towards the end of an interview with a suspect in in order to challenge their accounts.”

(GO)

“I give them the opportunity to account for their actions. I will then bring out that that CCTV because it’s my time to then trip them up.”

(TS)

They also describe varying disclosure of DE by attribution strength. In cases of strong attribution (e.g., personal devices, BWC footage), DE may be presented early (“Officers often show digital evidence at the start in crimes with strong attribution”, MS). This mirrors principles that favour phased or later disclosure to surface inconsistencies (Hartwig et al., 2005; Oleszkiewicz & Watson, 2021) and extends Polman et al. (2024) by showing how, in practice, officers actively calibrate disclosure not only by timing but also by the perceived attribution strength of DE. However, participants “don’t always hold it back” and believe that sometimes “it’s better to get the evidence in straightaway and confront the suspect with it” (BL).

Our data shows rich accounts of perceived suspect reactions to DE. Participants report that the presentation of DE frequently contributes to early guilty pleas, describing it as compelling and difficult to contest. They reflect that the way suspects react to DE can be linked to the strength of attribution and the timing of presentation:

“Some seasoned criminals are emotionless, but others panic, sweat… and start a sequence of mistruths.”

(BL)

“[If] the attribution is quite strong… they will go ‘no comment’. ‘No comment’ because there’s nothing they can say. Or they will start talking.”

(NC)

Sex offenders demonstrate particularly strong emotional responses when confronted with DE, extending the literature on confessions in the digital domain (Kassin & Gudjonsson, 2004). For example, IP notes that suspects in indecent image investigations often show relief when confronted with DE. Participants also remain aware of post-custody risks, balancing their investigatory goals with a duty of care:

“It’s a relief for them… they just bare all to us… they become quite emotional and you end up feeling sorry for them. They’re our biggest risks, more likely to harm themselves, the suicide rate is quite high in those cases.”

(IP)

#### Subtheme: Digital Devices and DE in Victim-Centred Interviews

Beyond suspect-focused investigative interviews, participant accounts shows that digital devices and DE plays a vital role in victim interviews, particularly in rape cases and other serious sexual offences. Participants report that “victim interviews feed into offender interviews” (CL) and evidence collection plans. Victim narratives help build timelines, attribute device ownership, and shape how officers pursue DE against suspects. The use of VRIs is also a common practice in the context of vulnerable witnesses such as victims of sexual assault, domestic abuse, as well as children. These recordings help capture non-verbal disclosures in sensitive interviews:

“The VRI is the substantial piece of evidence… children will point to places which is important because that’s captured on the video…”

(TS)

“Usefulness of that can’t be overstated, especially when dealing with vulnerable victims that don’t like putting pen to paper.”

(BL)

“He punched me on my head like this… you can see the amount of force… you can see the bruise as well.”

(TS)

Victims may also capture important evidence themselves, such as injuries or abusive messages, using their own devices:

“It’s not uncommon for a victim to photograph their injuries or for an abuser to film their abuse and send it to them.”

(BL)

A key role participants play is in safeguarding victims. This includes giving technical advice on how to protect their digital devices, accounts, and home environments. Participants often assist victims in recovering or providing their own DE in a less intrusive and more empowering way:

“We’ll guide them on extracting Facebook or Apple archives.”

(NC)

“We give safeguarding advice, like securing routers, changing passwords, locking down Alexa devices, preventing abusers from controlling smart homes.”

(WS)

Where needed, officers also “triage a phone or search a property for bugging devices” (NC) (e.g., hidden microphones or trackers), and “offer a digital search facility for victims of stalking… searching homes, cars, devices” (SK). Throughout DE recovery with victims, officers work to reassure, protect dignity, and explain procedures clearly under the College of Policing update made in 2021 to reflect GDPR for data:

“Sometimes we’ll just ask victims to screenshot messages and email them, rather than seizing the phone… unless it’s a serious offense.”

(BL)

“We have to bear in mind human rights and interrogation of devices and collateral intrusion.”

(GO)

Participants recognise that “it’s a daunting process… make them live the worst moment in their lives” (TS), emphasising their role in minimising harm, not re-traumatising victims:

“We remind victims we’re here to support them, represent them, and help them achieve closure.”

(BL)

“We’ll sit down with victims… and explain things in a way they understand… so they feel confident and safe.”

(SK)

### 3.4. Theme Four: DE in the Courtroom

Most of our participants report attending court to present DE or provide testimonies with regard to DE. They note that many legal professionals, particularly barristers and judges, lack digital literacy. Because of this gap, DE is rarely challenged in court as lawyers often do not have the technical expertise to question its admissibility, often wanting a simplified answer and “undervaluing” DE (JK):

“Defence barristers don’t understand how difficult it is to crack a phone.”

(MS)

“Digital evidence is particularly vulnerable because the judiciary often don’t understand it either… There’s no real check and balance… Most digital evidence that is accepted, it’s not really challenged.”

(NC)

Participants also observed that judges and juries struggle when it comes to interpreting digital forensic processes such as signal data and device logs, which is concerning given that barristers “lead the show” (JK).

“Especially with the cell site evidence… juries don’t get it. You can see people’s eyes just glazing over.”

(SK)

This can result in missed opportunities and dilution of the complexity of DE. Instead, barristers scrutinise chain of custody and procedural handling instead.

“The defense don’t argue about the evidence that you’ve got. What they argue about is your processes in relation to recovering that evidence.”

(RP)

Participants emphasised that the adversarial nature of courtroom presentation often masks not only the complexities but the gaps and limitations of DE. Much of it, they warned, would not withstand rigorous cross-examination, pointing to issues such as incomplete extractions (“Even on the 50 GB extracted, people don’t have time to review it all”, NC), and poor disclosure of what was not analysed:

“More often it doesn’t get investigated, because they haven’t got the time and the resource to be able to do it.”

(RP)

“I would be lying to say if even if I went to work for the defence at the minute, I will probably get quite a bit of digital evidence kicked out of court just by asking four or five little questions that would undermine the data in its entirety.”

(NC)

As we have begun to highlight in the previous theme, courts do not engage with raw digital data directly; they rely on its interpretation. Our findings reflect that what is presented in court is not the data itself, but one’s interpretation and explanation of it. Even high-quality extractions can be weakened by poor or biased interpretation. An adversarial court system means that different experts can explain the same evidence differently, which highlights the importance of expert interpretation:

“One court might hear a defence expert challenge GPS data, another court might see it upheld. You know, in the next, in the next courtroom, they might be supporting it, but it depends which side on the fence they sit upon.”

(JK)

This then puts the pressure on police officers attending court. DB described the experience as emotionally taxing:

“I’ve done it many times and to this day I still get like a slight anxiety attack before I go.”

(DB)

## 4. Discussion

This study examines how police officers in England and Wales perceive, understand, and use DE across four key themes. Participants also reflected on the future trajectories of DE (e.g., encryption, deepfakes, digital revisitation of crime scenes), but this lies beyond the scope of the present paper and will be reported elsewhere.

### 4.1. Sense-Making and Handling of Digital Devices and DE in Investigations

Research shows that early detection of DE in crime scenes is essential, as it ensures seizure and preservation of what can be used in investigative interviews (Holt et al., 2020). However, as our findings show, the ability to identify (and therefore secure) digital sources varies amongst our participants. Some participants struggle with basic definitions of “DE” and lack confidence when it comes to interpreting it. This lack of clear definitional understanding has a direct impact on interview preparation (e.g., who to confront, with what digital exhibits) as well as how confidently they can approach and question suspects, as will be expounded below (Weick, 1995). This further emphasises that the use of DE is interpretive, as shaped by the meaning officers construct DE for themselves and for suspects.

Of note, participants reference forms of DE generated by law enforcement themselves, such as BWCs, LFRs, and VRIs. VRIs are used to support disclosures from vulnerable victims and witnesses, including children (Ministry of Justice, 2023). They are particularly valuable because they can capture information through a dynamic visual format (e.g., the level of force involved in a punch) and support vulnerable victims or witnesses in describing difficult experiences.

This theme also highlights the critical features of DE: its volatile, mobile, and ephemeral nature (Henry, 2009). Participants in this study describe similar challenges in retrieving data (e.g., from encrypted apps, cloud material, router logs, and time-sensitive CCTV) to those described in Jones and Brookman (2024b) who examined the challenges in retrieving mobile phone data in homicide investigations. They frequently have to make golden hour decisions, where there is an urgency to preserve volatile data. In several cases, officers had to act quickly to safeguard such data to avoid its loss by deviating from protocol. Klein and Calderwood’s (1996) theory of recognition-primed decision-making can help explain this “Ronin-like” decision-making behaviour as seen in our participants, where they justified their deviations. Findings also reflect Simon’s (1972) concept of bounded rationality, where officers need to make decisions under time pressure as well as with limited technical knowledge. They describe prioritising digital devices based on shortcuts such as the type of crime and device familiarity, sometimes disregarding digital leads altogether.

### 4.2. The Interpretation and Reliability of DE

Like other forms of forensic evidence, this study shows that DE is actively interpreted through officers’ cognitive lenses. Its value depends not only on the data but also on how it is reviewed and understood. Participants note that misunderstandings and errors can happen in several key points of the investigative process, such as digital logs being misunderstood or overlooked due to time pressure. This echoes past studies that show DE is not neutral nor self-explanatory (Brookman & Jones, 2022; Wilson-Kovacs et al., 2023). As described elsewhere, these handover points (where DE passes between officers, DFIs, and prosecutors) are both labour-intensive (Brookman et al., 2020) and error-prone (Brookman et al., 2020; Dror, 2025). The meaning of DE can shift at each of these handover points, from interview room to courtroom, producing what Elzinga (1997) refers to as epistemic drift. Here, the loss or alteration of meaning can undermine DE, potentially leading to miscarriages of justice (McCartney et al., 2024). Epistemic drift in this context is not a single point of data loss or alteration, but a cumulative process whereby the meaning of DE gradually changes as it moves between officers, digital forensic specialists, and court personnel (Dror, 2025; Elzinga, 1997).

A further challenge that participants raise comes from digital content authentication in the age of generative AI. The “liar’s dividend” (Chesney & Citron, 2019) means that suspects can plausibly deny real evidence by pointing to the possibility that it is deepfaked or doctored. CCTV and recordings are now more easily contested, especially when officers lack the training to spot real from fake digital content and when lawyers struggle to verify, explain, and defend it. As a result, generative AI therefore increases the risks of epistemic drift, creating new vulnerabilities in attribution and authentication across the investigative lifecycle.

### 4.3. Use of DE in Investigative Interviews with Suspects and Victims

This theme (and subtheme) uniquely explores how DE is used during investigative interviews with suspects and victims. While investigative interviewing is a well-established field, studies have yet to examine how DE is used within these interviews (Holmes, 2024). We find that participants tailor when they disclose DE based on the interview phase, its attribution strength, and suspect behaviour.

In England and Wales, police officers operate under the PEACE framework (Shawyer et al., 2009). While the framework provides structured guidance, it allows officer discretion as to when and how to introduce DE during investigative interviews. Our study found that attribution strength plays a central role in the timing of DE presentation. Where attribution was weak (e.g., ambiguous cell site data), participants use caution. In cases where attribution is strong (e.g., synced devices, unique metadata, face-matching), participants report confronting suspects early and directly. These findings align with experimental work that show the advantages of strategic and phased disclosure (Hartwig et al., 2005; Oleszkiewicz & Watson, 2021; Polman et al., 2024) and extends them into the domain of DE.

Our findings demonstrate that the disclosure of DE is not only a matter of timing; it also depends on how it is translated into ways that are easy for suspects to understand during investigative interviews (e.g., when digital exhibits are shown on laptop screens, PDFs, printouts, and evidential clippings from large datasets as exhibits). Officers must make sure that complex DE is communicated in a manner that both interviewers and interviewees can meaningfully understand and engage with.

It goes without saying that effective interviewing involving digital devices and/or DE requires fluently understanding concepts such as the provenance and attribution of DE, as much as challenging a suspect’s account. The impact of disclosing DE is diminished if suspects cannot comprehend technical details; if officers are unable to explain DE clearly, they are less able to use it strategically. We find that officers sometimes struggle to frame the right questions because they have difficulties interpreting reports and data handed over by digital forensic specialists.

It is interesting to note in our sample that when DE is shown to suspects, they often either admit their guilt or respond with “no comment”. This suggests that suspects view DE as a powerful type of evidence that frequently results in confessions, especially in cases of sexual offenses. Importantly, this echoes in a digital context the assertion of Moston and Engelberg (2011) that suspects’ perception of evidence is important. Importantly, participants in our sample acknowledge the risks of so-called “digital strip searches”, and report seeking only what is required for a reasonable line of enquiry (ICO, n.d.; NPCC, 2024). Overall, these rich accounts emphasise a trauma-informed approach for both victims (HMICFRS, 2022) and suspects alike (Gordon Smith, 2018; Tudor-Owen et al., 2023).

### 4.4. Challenges of DE in the Courtroom

This theme illustrates that by the time DE reaches the courtroom, it is no longer raw data but an interpretation as shaped by officers, DFIs, and court actors alike. Building on research that showcase the adversarial nature of UK courts (Jones & Brookman, 2024a), our findings show that the evidential value of DE in the courtroom is highly dependent on its interpretation and communication. In this process, important details can be lost, misunderstood, misused, or blindly accepted without much challenge (Dror, 2025).

Participants in this study identify a digital literacy gap amongst barristers, judges, and jurors, echoing past studies (Casey, 2019; Jones & Brookman, 2024a; Wilson-Kovacs et al., 2023) and further warns of the impact of this. Courtroom actors either undervalue DE (because it feels confusing) or over-trust it (because it is presented as “objective”). The challenge we highlight here is not only that (1) DE often enters court with little resistance; but that (2) its provenance, limits, and attribution is also poorly understood once admitted. Participants state that barristers exploit these gaps, while the burden falls on officers acting as professional witnesses. Furthermore, our findings point to new risks around AI-generated material and deepfakes, raising further questions of its attribution and authenticity in court.

## 5. Future Directions

While the importance of timing has long been highlighted in disclosure research (Hartwig et al., 2005; Oleszkiewicz & Watson, 2021; Polman et al., 2024), our study demonstrates that DE introduces additional demands and pressures in relation to its interpretation, translation, and susceptibility to emotional reactions in suspects. These findings point to several areas for development.

First, our research indicates that digital literacy must be treated as a core policing competence in order for frontline officers to recognise, understand, and engage with DE meaningfully and confidently. What is evident from this study is that interpretive and translation skills for DE are equally important, as officers must translate complex digital reports into clear explanations for suspects, victims, witnesses, prosecutors, and juries. Findings suggest a shift towards digitally native investigations, where frontline officers can perform basic digital tasks on their own, rather than always relying on or deferring to digital forensic labs. This includes tasks such as being able to clip CCTV, exporting telematics data (e.g., vehicle location and speed), and preserving social media posts (GovNet, 2025; Police Digital Service, n.d.).

Second, training should focus on interpretation and communication of DE, as effective disclosure of DE is not only a question of when evidence is revealed, but also of how it is translated and communicated in ways that make sense to both interviewers and interviewees. Preparing digital exhibits in simple, digestible formats is part of this skill. Without this capability, suspects may exploit an officer’s uncertainty and disclosure of DE may have less of an impact. This training also makes it more likely that DE is understood by lawyers, judges, and juries later down the line.

Third, our findings indicate that some suspects show distress and pose risks of self-harm when confronted with sensitive DE, particularly in child sexual offenses (e.g., offences involving indecent images of children). This reflects the unique intimacy of DE, highlighting the need for clearer guidance and training for officers in managing acute distress and safeguarding suspects in these interview settings (Tudor-Owen et al., 2023; Vaughan et al., 2024), while managing their own emotions when conducting these interviews (Gordon Smith, 2018).

Fourth, our findings indicate that epistemic drift in DE is particularly vulnerable during the handover points in the investigative lifecycle, in part due to poor communication between key actors along the way. Currently, most communication between the many units and actors involved is mostly informal or disjointed, often taking place over the phone or emails, or through ad hoc briefings. Officers frequently receive technical reports that they are unable to fully interpret, while DFIs may not have full case context to prioritise their analyses. While a few forces communicate via internal dashboards or ticketing systems, these are not national standards. A centralised dashboard integrated with existing police case management and record systems could streamline workflows, record informal exchanges systematically, and reduce knowledge silos. By enabling different actors along the investigative cascade to share case information, requests, and digital queries in one interoperable space, such systems could improve DE quality, mitigate errors, and build trust. This aligns with the UK’s Digital First programme to standardise case file sharing with the CPS (Digital Policing Portfolio, n.d.).

Last but not least, studies on forensic-specific large language models (LLMs) indicate that they may help officers at critical stages of an investigation, such as analysing and summarising large volumes of DE, spotting patterns between devices or accounts (Yin et al., 2025), and pre-interview planning (Järvilehto et al., 2025; Santtila et al., 2025; Sun et al., 2025). Participants often find it challenging to locate relevant information within vast volumes of digital data (“Looking for a needle in a haystack”, CL); LLMs are designed precisely for this type of large-scale pattern recognition and analysis (Yin et al., 2025). At the same time, they also raise new epistemic challenges. Given that officers are already facing difficulty in interpreting DE, our findings highlight the need for these tools to be critically designed with transparency, validation, and auditability (Garrett & Rudin, 2024). In light of this, these systems should be governed and implemented to support officers, rather than replace, their interpretive expertise, thereby preventing further epistemic drift.

## 6. Limitations

One limitation of this study pertains to the diversity of the sample. Just two participants identify as female, reflecting a broader gender imbalance in digital forensics (Wagstaff & LaPorte, 2018). Furthermore, this study’s participants all identify as cisgender, meaning minority viewpoints are underrepresented. Findings might therefore not fully capture the full range of experiences found within the policing workforce. While every effort was made to involve participants from a range of forces across England and Wales, this invariably limits the applicability and the generalisability of the findings to all UK policing contexts (and beyond). Future research should aim to recruit more inclusively and broaden recruitment across a wider range of forces and departments, within and beyond the context of England and Wales, to ensure a fuller understanding of digital policing experiences.

## 7. Conclusions

DE now features in practically every criminal investigation, and this study provides critical insight into its human dimension. Through a holistic, investigative lifecycle perspective, we demonstrate the complexity of the interpretive work that officers must do. The usefulness and utility of DE is actively constructed through officers’ understanding, interpretation, discretion, and communication. This spans from making decisions about what to seize at crime scenes and translating technical reports for use in interviewing to communicating in court with lawyers and juries alike. This work is seldom recognised, nor is it supported by training. Importantly, this study also shows how meaning can be created, lost, or altered as DE moves through multiple actors in the investigative cascade. Findings extend experimental work on the benefits of phased disclosure (Hartwig et al., 2005; Oleszkiewicz & Watson, 2021; Polman et al., 2024) into the digital domain. They demonstrate that the strategic use of DE involves not only the timing of disclosure, but also the interpretive and communicative work required to give DE its meaning. Furthermore, suspects’ reactions to DE can be particularly strong in child sexual offences cases. This indicates that digital justice depends as much on the human elements as it does the technical processes, and reforms should be made to: (i) treat digital literacy as a basic competency for all officers, (ii) offer more training on how to interpret, translate, and explain DE, (iii) embed trauma-informed approaches when interviewing suspects in sensitive cases, (iv) create standardised frameworks and communication systems to reduce epistemic drift, where meaning is lost or changed at handover points, and, lastly, (v) implement decision-support tools to help officers at key points in investigations.

## Figures and Tables

**Table 1:** Key differences between DE and traditional evidence.

Characteristic	Digital Evidence	Traditional Evidence
Nature	Intangible (binary form and stored electronically)	Tangible
Volatility	High, easily lost/altered	Low, physically persistent
Mobility	High, easily transferred	Low, fixed location
Data types	Varied (e.g., text messages, GPS, logs)	Physical (e.g., fingerprints, fibres)
Storage medium	Devices, cloud	Physical containers, bags
Presence of metadata	Often includes metadata—information about how, when, and by whom a file was created or modified	Does not usually come with metadata
Replicability	Can be duplicated exactly without degradation	Cannot be exactly duplicated

## Data Availability

Supporting data are publicly available on https://osf.io/eapsh/?view_only=13983b05998445478ef3a30759ec3f04.

## Supplementary Materials

The following supporting information can be downloaded at: https://osf.io/eapsh/?view_only=13983b05998445478ef3a30759ec3f04.

## Abbreviations

The following abbreviations are used in this manuscript:

DE	Digital evidence
DMI	Digital media investigators
DFI	Digital forensic investigators

## Appendix A

### Main Author’s Reflexive Statement

My background in investigative psychology no doubt shapes how I would have approached this study, which sits in the crossroads of psychology and digital forensics. Here I acknowledge the influence of my own experiences, knowledge, and background in investigative psychology on data interpretation. My background gives me a useful start in understanding the literature around decision-making, investigative interviewing, and cognitive biases. However, it brings with it potential biases and blindspots in digital forensic processes. For example, I recognise that I could have overlooked some important aspects of digital forensics. To reduce these potential biases and blindspots, I took several proactive steps throughout the project. After familiarising myself with a wide range of literature in digital forensics, I brought in a co-author with expertise in the field of digital forensics (RM), and two other co-authors (CD and RB) from outside the field to bring in different perspectives. Together as a team, we regularly had collaborative, open discussions. Having RM in the team strengthened this reflexive approach, contributing to a better understanding of the digital elements in criminal investigations and helping me continuously reflect on how I interpret the data as shaped by my background.
